# The diagnostic value of the lumbar infusion test to predict symptomatic improvement after shunting for normal pressure hydrocephalus. A meta-analysis

**DOI:** 10.1007/s00701-025-06591-8

**Published:** 2025-06-30

**Authors:** M. W. T. van Bilsen, V. Volovici, S. Arts, L. van den Abbeele, H. D. Boogaarts, R. H. M. A. Bartels, E. J. van Lindert

**Affiliations:** 1 Erasmus Medical Center, Department of Neurosurgery, Dr. Molewaterplein 40, 3014 GD Rotterdam, The Netherlands; 2 Department of Radiology, Albert Schweitzer Hospital, Dordrecht, The Netherlands; 3 Department of Neurosurgery, Radboud University Medical Center, Nijmegen, The Netherlands; 4 Center for Complex Microvascular Surgery, Erasmus MC University Medical Center, Rotterdam, The Netherlands

**Keywords:** Lumbar infusion test, Normal pressure hydrocephalus, Diagnostic value, Specificity, Sensitivity, Shunting

## Abstract

**Background:**

The aim of this meta-analysis is to determine the diagnostic value of the Lumbar Infusion Test (LIT) to differentiate between patients suffering from normal pressure hydrocephalus who will benefit from CSF shunting and those who will not.

**Methods:**

A systematic search was performed in Ovid MEDLINE to identify RCTs or observational studies that evaluated LIT for predicting shunt responsiveness. Sensitivity and specificity values were pooled and Bayesian meta-analysis was performed.

**Results:**

The Resistance to outflow (Rout) sensitivity was 76.9%; 81.6% and 36.6% for a Rout cut off of respectively 12, 14 and 18 mmHg/ml/min. Specificity rose with higher Rout cut off and was 34.0%; 37.2% and 78.0% for a cut off of respectively 12, 14 and 18 mmHg/ml/min. The negative predictive value was low for each cut off and was at most 33.3%.

**Conclusion:**

The LIT proves poor negative predictive value and appears an ineffective tool in the prediction of non-response to a shunting procedure. Therefore, in its present form and based on its current parameters, it cannot be used as a test to exclude patients from shunt implantation.

**Supplementary information:**

The online version contains supplementary material available at 10.1007/s00701-025-06591-8.

## Background

Normal pressure hydrocephalus (NPH) was first described in 1965 by Hakim and Adams [11]. Patients with NPH present clinically with a triad of symptoms i.e., urinary incontinence, dementia and gait disturbances. Radiologically, ventriculomegaly is visible on imaging studies. Its prevalence is estimated to be 1.3–3.7% among individuals over the age of 65 years [1, 19].

NPH is an important differential diagnosis of neurodegenerative diseases, since the condition can improve with cerebrospinal fluid (CSF) shunting [10, 12, 17, 28]. However, CSF shunting is an invasive procedure with possible complications such as intraparenchymal or subdural hematomas, epilepsy, shunt infection and need for shunt revision [10]. Therefore, in order to select patients who benefit from shunt placement, a lumbar infusion testing (LIT) is used in some centers [9, 24, 28].

LIT is based on the principle that patients with NPH have changes in cerebrospinal fluid (CSF) hydrodynamics compared with patients without NPH [24, 27]. The test measures lumbar CSF pressure while infusing fluid intradurally. However, the true diagnostic value of LIT is unknown, especially in excluding patients from shunt implantation. Few studies have pooled diagnostic values of LIT as part of a review article of multiple diagnostic modalities, stating sensitivity and specificity and consider LIT as a valuable option in the diagnostic workup of NPH. However, the reported poor negative predictive value in individual papers rases concerns about its usefulness in excluding patients from shunt implantation. Until now no diagnostic accuracy analysis has been performed.

The purpose of this meta-analysis is to determine the negative predictive value of the LIT to withhold patients with normal pressure hydrocephalus from a shunting procedure since it would not be beneficial for them.

## Methods

### Lumbar infusion test

**Fig. 1:**
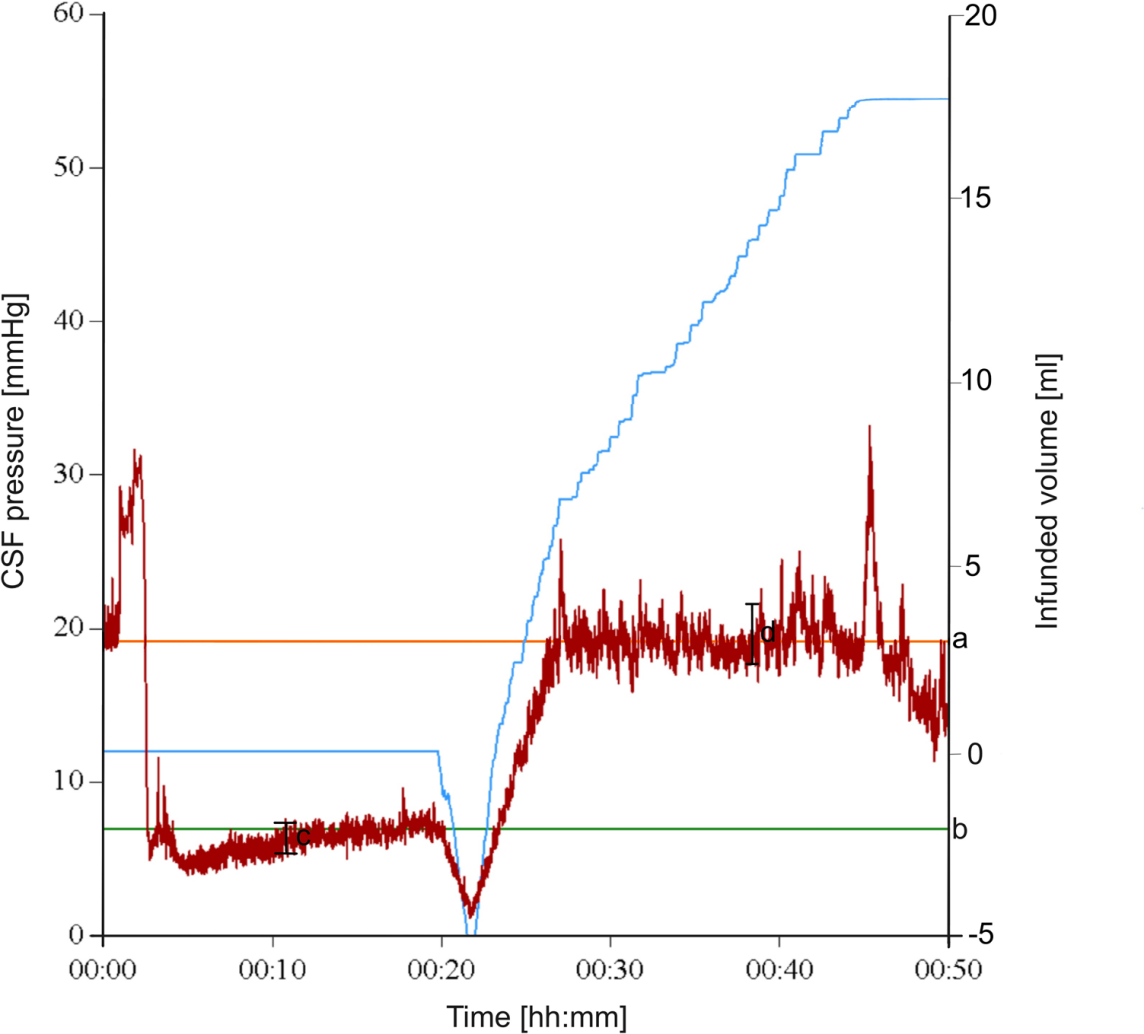
Example of a LIT measurement reading. a = plateau pressure (mmHg); b = initial pressure (mmHg); c = initial pressure amplitude (mmHg); d = plateau pressure amplitude(mmHg); Rout = (a-b)/infusion rate. First peak occurs because of a lumbar puncture in a sitting position. Dip at 20 min occurs due to aspiration of CSF by the registered nurse before the initiation of infusion. Blue line, infused volume in ml; red line pressure in mmHg

Lumbar infusion tests are based on the principle that, under normal physiological conditions, an increase in intracranial pressure via infusion of fluid leads to an increased absorption of CSF, which will lead to a new equilibrium visible as a plateau in a pressure curve. Among others, failure to achieve this equilibrium, or increased pressure when this equilibrium is reached, may indicate disturbed CSF circulation and thus hydrocephalus. This also applies if the pressure was not increased before infusion of fluid, thus indicating normal pressure hydrocephalus. However, the pathophysiology of this disturbed circulation remains unknown to date. Different parameters of LIT, as plateau pressure (mmHg), initial pressure (mmHg), initial pressure amplitude (mmHg), plateau pressure amplitude (mmHg), amplitude during infusion (mmHg) and Resistance to outflow (Rout, mmHg/ml/min) can be obtained from the graph which results from plotting the pressure against infused volume and time (Fig. 1). Rout is thus a normalized parameter and calculated by dividing the pressure difference of the plateau phase and the starting pressure by the infusion rate. A normalized index makes differences in infusion rate irrelevant and makes results between different subjects or cases comparable [27]. Previous authors have also hypothesized that patients with NPH may have higher CSF pressure amplitudes than normal subjects ([14, 22] and this could cause ventriculomegaly.

### Research protocol

The protocol for this systematic review was registered on PROSPERO (CRD42020210345) and is available in full on the *Prospero, University of York* website [23].

The study was carried out according to the Preferred Reporting Items for Systematic Reviews and Meta-Analyses (PRISMA) guideline [21].

### Search strategy

The search strategy was developed together with a library search specialist and contained free text words, based on conceptual similarity, determined by consensus between all authors and a conversion of all words into MeSH-terms. The search was performed in Ovid MEDLINE from the date of the first publication until June 2024. Clinicaltrials.gov was consulted to search for ongoing studies. Additional records were identified through backtracking of the reference lists of all relevant articles.

### Inclusion criteria

A study was eligible for inclusion if it was peer reviewed and written in English, described adult patients with clinical suspicion of NPH who underwent a lumbar infusion test, described at least one of the follow parameters: plateau pressure (mmHg), initial pressure (mmHg), initial pressure amplitude (mmHg), plateau pressure amplitude (mmHg), amplitude during infusion (mmHg) or Resistance to outflow (Rout, mmHg) and reported the clinical outcome after the implantation of a shunt or after the institution of a conservative policy. Only Randomized controlled trials, retrospective and prospective cohort studies were eligible for inclusion. Furthermore, studies were included in the meta-analysis if its outcomes were quantitatively measured and sufficient outcome numbers were available to be pooled.

### Outcome measures

The primary outcomes were the pooled sensitivity and specificity for the LIT to shunt response. Improvement after shunt implantation was defined as any improvement on either the NPH scale, MMSE, mRS or walking tests, as defined and reported by the investigators. Articles with only baseline descriptive data of subjective outcomes were excluded from the meta-analysis. NPH scale was defined as a combined score of clinical assessments in the domains gait, continence and cognition.

### Data extraction

Each study was assessed by two investigators (MVB, SA). When inconsistencies were identified, articles were discussed with a third investigator until consensus was reached. The same investigators extracted study data into a data extraction form that included general study details, number of patients, time to follow up, type of outcomes, effect sizes and confidence intervals for primary and secondary outcomes, dependence of shunt implantation of LIT, infusion test protocol and Rout. Lastly, the risk of bias was assessed by using the Quality Assessment of Diagnostic Accuracy Studies-2 (QUADAS-2) [31].

### Statistical analysis

RevMan (The Cochrane Collaboration, Copenhagen, Version 5.4.1) and MetaDisc (Ramon y Cajal Research Institute, Madrid, Version2.0) [33] were used to calculate sensitivity, specificity, negative predictive value and positive predictive value in the meta-analysis. Summary sensitivity and specificity and their 95% Confident intervals were obtained with bivariate modeling. The hierarchical summary receiver operating characteristic curve (HSROC) model was used in R (The R project for statistical computing, Vienna, version 3.6.1) because of different reference diagnostic tests used in the various studies. We then performed a Bayesian diagnostic meta-analysis using the packages “meta4diag”, “bamdit” and “mada”. This approach assigns each study a random component D, representing the study effect associated with the diagnostic discriminatory power and a random effect S, which quantifies the variability produced by patients’ characteristics, study design and diagnostic setup, that may produce a correlation between the true and false positive rates observed. This latter approach is necessary when different thresholds are used and the results are likely sensitive to diagnostic settings.

Using weakly informative priors for the equation hyperparameters and 10 000 simulations we calculated the posterior distribution of both sensitivity and specificity and represented these visually by plotting the predictive surfaces at the 50, 75, and 95% quantiles. We then evaluated the studies for outliers and performed sensitivity analyses by leaving these out. A further sensitivity analysis was performed by replacing the binomial model with a normal distribution with a binomial model with scale mixture distributions.

We calculated the pooled sensitivity, specificity and negative predictive values (NPV) of individual parameters of the LIT and of their combinations. If insufficient data are available we analyzed the most used cut-offs. A sensitivity analysis was performed, by calculating the diagnostic parameters for only studies in which the indication for surgery was independent from the LIT result, as this might have confounded outcomes.

## Results

### Included publications

Supplementary Fig. 1 shows a PRISMA diagram of included studies. The search yielded a total of 215 articles of which 10 articles could be included in the final analysis.

### Characteristics of included studies

Details are provided in Table 1. Mean age of patients varied per study from 65 to 77 years. Eight studies used Rout as indicator of LIT, 1 study combined this Rout with the CSF amplitude during lumbar infusion as a predictor for shunt responsiveness and one study focused exclusively on CSF amplitude. One study used the delta amplitude and one study the slope until reaching the plateau phase to predict shunt response. Five studies had a prospective design, and 5 studies had a retrospective design. The number of patients included per study ranged from 17 to 127. The Time to follow up varied from 3 months till 12 months. All studies used a NPH-scale to define positive result after. Two studies added mRS and one study added a reaction time test as secondary outcome. Five studies used LIT results to indicate surgery. In the 5 remaining studies, surgery was independent of LIT results.

**Table 1:** Characteristics of studies included in the meta-analysis

	N	Design	Mean age (range)	LIT measure-ments	Definiti-on of Posi-tive LIT*	Time to last FU (months)	Outcome measure	Shunting depending on LIT measurements
Boon et al., 1997(20)	95	P	74 (50–85)	Rout	Rout > 12 Rout > 15 Rout > 18	12	1, 3	No
Eide et al., 2010(26)	45	R	72 (37–85)	PinA, PplA	CSFampl during lumbar infusion </> 4	12	1	Yes
Junkkari et al., 2019(33)	48	R	72 (n/a)	Rout	Rout > 12	3	1	Yes
Kahlon et al., 2005(25)	55	P	74 (43–85)	Rout, Ppl, Pin, PplA	Ppl > 22 Rout > 14 Rout > 18	6	1, 4	Yes
Raneri et al., 2017(34)	52	R	72 (45–85)	Rout, Pin	Rout > 14	12	1, 3	Yes
Sorteberg et al., 2004(21)	17	P	65 (36–80)	Rout	Rout > 12	9	1	No
Wikkelsø et al., 2013(19)	115	P	70 (30–87)	Rout	Rout > 12 Rout > 18	12	1, 3	No
Otero-Rodriguez et al., 2023	110	R	77 (> 60)	Rout, SRP	Rout > 12	12	1	Yes
Van Bilsen et al., 2022	38	P	72 (68–76)	Delta amp	Delta amp > 0,20/0,25/0,30/0,35	6	1	No
Gregers Hasselbalch et al., 2023	127	R	74 (55–87)	PinA, PinA/Pin; Rout	Rout > 11/16/19; PinA; Pin	2	1	No

N, number of patients included in the analysis; R, Retrospective; P, prospective; Rout, resistance to outflow in mmHg/ml/min; Ppl, plateau pressure in mmHg; SRP, Slope until reaching the plateau; Delta Amp, Delta Amplitude in mmHg; FU, follow up in months; 1, a combined score of gait, incontinence and cognitive tests; 2, Mini Mental State Examination; 3, modified Rankin Scale; 4, reaction time test; Pin, initial pressure (mmHg); PinA, initial pressure amplitude (mmHg); PplA, plateau pulse-pressure amplitude (mmHg);

^*^ outcomes are available for all different options given

### Outcomes

Sensitivity, specificity and negative predictive value are displayed in supplementary appendix 1. For Rout the sensitivity was for all included studies 76.9%, 81.6% and 36.6% for a Rout cut off of respectively 12, 14 and 18 mmHg/ml/min. Specificity rose with higher Rout cut off and was 34.0%; 37.2% and 78.0% for a cut off of respectively 12, 14 and 18 mmHg/ml/min. For studies which did not indicate shunting based on LIT results [3, 13, 26, 32] specificity was 25% and sensitivity was 78% for Rout more than 12 mmHg/ml/min. For a Rout more than 18 specificity was 73% and sensitivity was 32%. The negative predictive value was low for each cut off and was at most 33.3%.

**Fig. 2:**
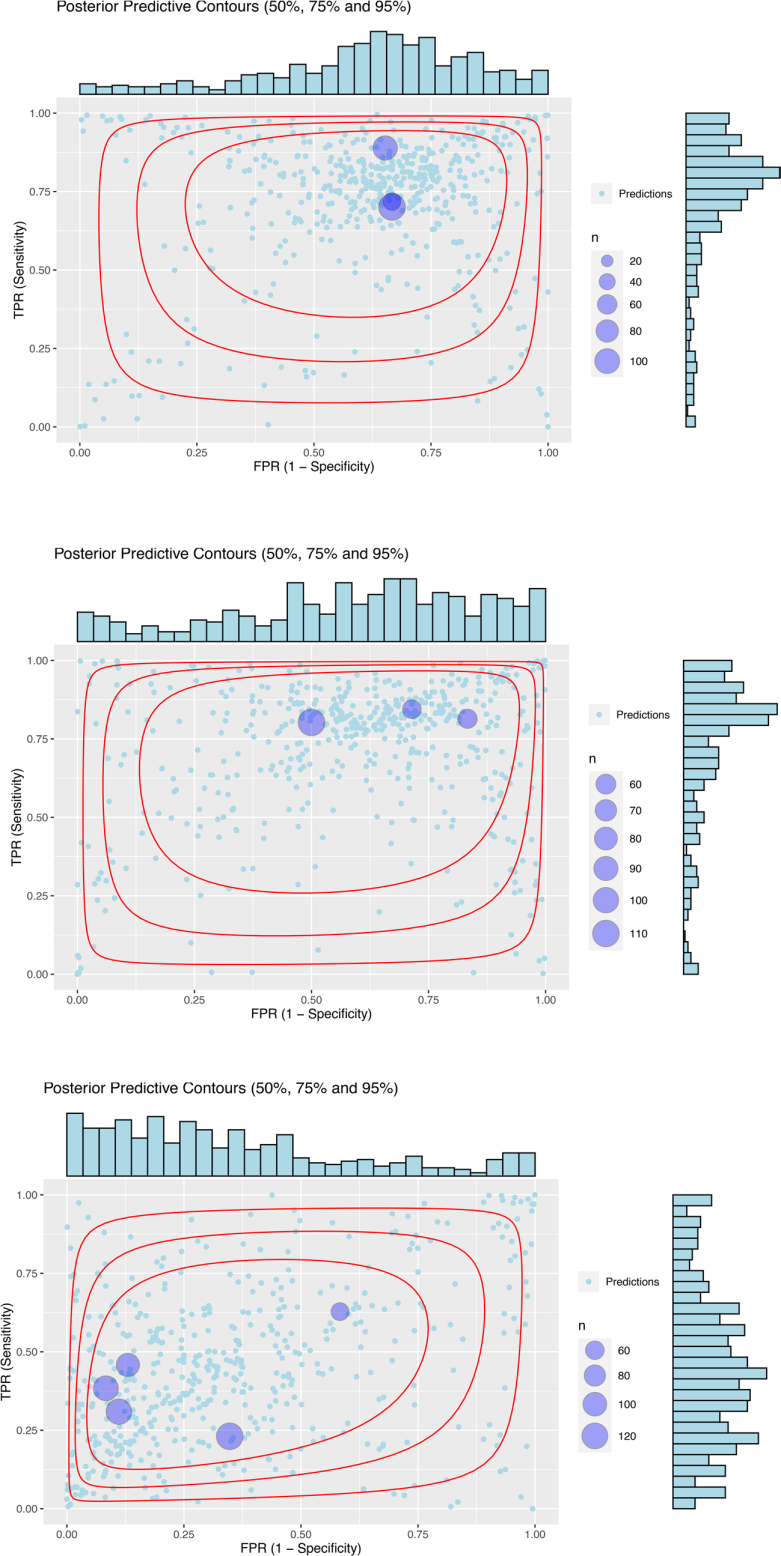
Results of the predictive surfaces of the Bayesian diagnostic meta-analysis with a bivariate normal distribution of LIT studies, random effect model and weakly informative priors. The plots show the results after 10,000 simulations. The blue points in the graph indicate the distribution of simulated studies. The red contours indicate the predictive surfaces of the 50%, 75% and 95% quantiles of these studies. The histograms show the posterior distribution of predicted values for these studies in terms of false positive rate (x-axis, above the graph) and sensitivity (y-axis, on the right side of the graph). TPR, true positive rate; FPR, false positive rate

The Bayesian analysis showed a consistent pattern of sensitivity and specificity (Fig. 2),

Three studies [7, 18, 20] explored the difference in Rout between shunt responders and non-shunt responders. Delwel et al. [7] preselected patients based on a Rout > 12, as a result of an earlier study by the same group [3] and which is included in this meta-analysis. This preselection did not lead to better overall shunting results.

### Risk of bias

All studies included in the meta-analysis all had a serious risk of bias (Table 2).

**Table 2:**
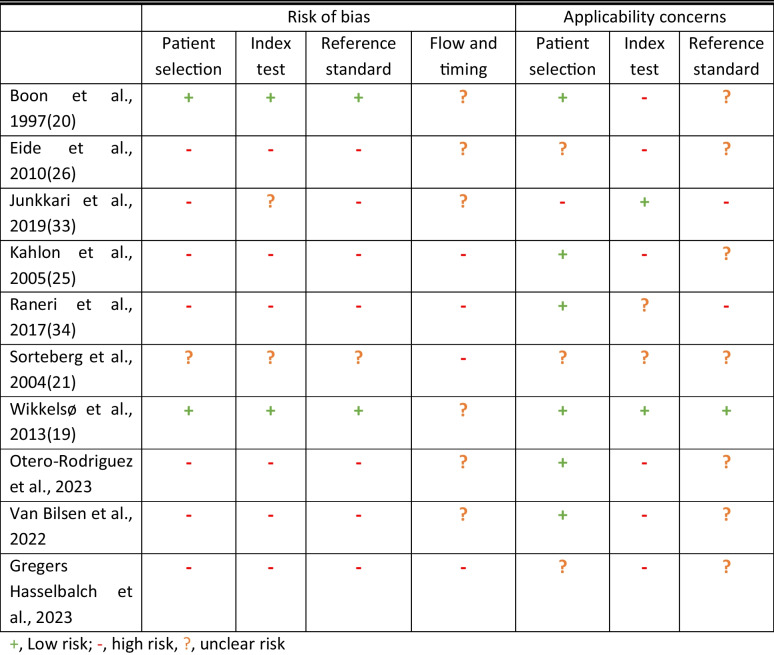
Risk of bias assessment according to QUADAS-2

## Discussion

The aim of this study was to determine the diagnostic value of LIT to predict shunt responsiveness in patients with idiopathic normal pressure hydrocephalus.

Statistical analysis showed poor negative predictive values of at most 33.3%. The results indicate that a LIT with high (Rout > 12 mmHg/ml/min)Rout Values in patients suspected for NPH are likely to have a positive clinical response after shunting, however negative Rout values cannot exclude a beneficial effect of surgery due to a high possibility of a false negative result Table 3.

**Table 3:** Pooled outcomes (point estimate (95% CI))

	Rout > 12	Rout > 14	Rout > 18
Pooled sensitivity	0,769 (0,706—0,825)	0,816 (0,750—0,871)	0,366 (0,318—0,415)
Pooled specificity	0,340 (0,212—0,488)	0,372 (0,230—0,533)	0,780 (0,686—0,857)
NPV (65% response rate)	0,262 (0,160—0,385)	0,333 (0,204—0,484)	0,234 (0,190—0,283)

*NPV*; negative predictive value, *Rout*; resistance to outflow in mmHg/ml/min

There are several factors that may influence these results. Selection bias might be one. The included studies include a selected population of patients that were eligible to receive LIT. All studies selected patients based on opening pressure on lumbar puncture, tap test, comorbidity or imaging characteristics. Furthermore, although all studies used a NPH scale to define improvement, some scale items are subjective. This might introduce interpretation bias. As the value of the LIT was based on this improvement and negative predictive values are based on the prevalence of NPH-scale improvement, this may influence results. Nonetheless, subjective improvement in such diseases is still important. This raises questions, however, whether or not a systematic positive bias and therefore low specificity is present in the studies.

Additionally, five out of ten included studies set the indication of shunt implantation based on LIT results leading to confirmation bias Hence, many patients with a negative Rout were not included in these studies. Nevertheless, pooling of studies in which LIT results were blinded or not influencing implantation [3, 13, 26, 32] did not yield better negative predictive value.

Time to follow up can influence shunt results, as some studies indicate that improvement after shunting is time-dependent and possibly also temporary [2, 16, 28]. Czosnyka et al. [6] investigated the impact of symptom duration on LIT parameters and improvement after shunting. They found diminished Rout in patients who experience symptoms longer than 2 years of time, but no dependency of shunt improvement on symptom duration. This may negatively impact sensitivity. Our pooled results show no association between the duration of follow-up and sensitivity or specificity rates. However the possible time dependency of improvement after shunt implantation can raise questions about the applicability of LIT before shunting, as it cannot exclude patients from shunt implantation and can cause delay.

Most studies included in this meta-analysis used Rout to predict shunt responsiveness. Other parameters that LIT yields are plateau pressure (mmHg), initial pressure (mmHg), initial pressure amplitude (mmHg) and plateau pressure amplitude (mmHg)(Fig. 1). Two studies included in the meta-analysis [8, 15] used other parameters than Rout to predict shunt responsiveness, namely plateau pressure, which is directly correlated to Rout, but also the CSF amplitude. Both studies did not find better diagnostic value of these parameters. Jacobsen et al. [14], studied ICP amplitude in patients with NPH and healthy controls. One study investigated delta amplitude in order to predict shunt response and did not find any correlation [30].

Some studies preselect patients based on a higher Rout for surgery [7, 18, 20]. However, this preselection did not lead to better overall shunting results. They found no differences neither in Rout nor CSF amplitude between shunt responders and non-responders. Malm et al. [18] also showed no diagnostic value of Rout values to distinguish shunt responders from non-responders. Meier et al. [20] compared outcome with Rout and found that an Rout more than 15 mmHg/ml/min to be correlated with more favorable outcome. These results are in line with the results of our meta-analysis.

The diagnosis of normal pressure hydrocephalus is still topic of debate. Previous authors even raised question about the existence of the disease itself and its reaction on shunt implantation [25]. Kazui et al. [17] compared in a randomized trial direct implantation of a lumboperitoneal shunt to a three months postponed implantation. Both groups showed a beneficial effect of shunt implantation after one year. Tisell et al. [28] performed a blinded and randomized controlled trial comparing the implantation of a ligated shunt to the implantation of an open shunt. They found an improvement in the open shunt group compared to the ligated group. Furthermore, they found improvement in the ligated group after removement of the ligation. One randomized controlled trial was registered [29] in 2012, but was terminated early, because of the observed benefit of treatment was larger than expected [4]. These results indicate the existence of a shunting effect in NPH patients. However Czepko et al. [5] followed a cohort of 27 patients who did not receive a shunt and had a mean Rout of 7.5 mmHg/ml/min. Surprisingly, after a mean follow up time of six months, no patients deteriorated and six patients even showed improvement. Hence there are strong suggestions NPH does exist as a separate entity, however knowledge pertaining to the disease is limited, which leads to a difficult to define patient population and limited treatment possibilities.

### Future of LIT in NPH diagnostics

So while it is evident that there is a category of patients that improves after shunting, this does not apply to all patients receiving the iNPH diagnosis. The challenge in future studies will be to define and diagnose the iNPH-shunt-responsive patient cohort. Unfortunately, LIT does have too many false negative results. Therefore we do not recommend the test as it is now to be used in order to indicate shunting or even more important, to withhold surgery from patients based on their LIT results. Future research endeavors should focus on improving NPV.

## Conclusion

The results of this meta-analysis indicate that the manner in which the lumbar infusion test has been used in scientific research so far, can fairly indicate a positive shunt response, but cannot predict negative shunt response. Therefore, in this form, it cannot be used as a test to exclude patients from shunt implantation.

## Supplementary information

Below is the link to the electronic supplementary material.

ESM 1 (DOCX 66.5 KB)

ESM 2 (DOCX 17.5 KB)

## Data Availability

No datasets were generated or analysed during the current study.

## Abbreviations

CSF: Cerebrospinal fluid
HSROC: Hierarchical summary receiver operating characteristic curve
ICP: Intracranial pressure
iNPH: Idiopathic normal pressure hydrocephalus
LIT: Lumbar infusion testing
MMSE: Mini-Mental State Examination
mRS: Modified Rankin Scale
NPH: Normal pressure hydrocephalus
Rout: Resistance to outflow
NPV: Negative predictive value
