# Supplementary material for: The diagnostic value of the lumbar infusion test to predict symptomatic improvement after shunting for normal pressure hydrocephalus. A meta-analysis
Source: Acta Neurochir (Wien). 2025 Jun 30;167(1):180. doi: 10.1007/s00701-025-06591-8 (PMC12209032; doi:10.1007/s00701-025-06591-8)
Supplement: Supplementary file 1 — (DOCX 66.5 KB) [file 701_2025_6591_MOESM1_ESM.docx]

**Supplementary appendix 1**

Records identified through database searching

(n=215)

Records selected for full text assessment

(n = 44)

Records excluded

(n = 171)

Full- text articles assessed for

eligibility

(n = 46)

Full-text articles excluded (n = 36)

Reason for exclusion:

- Study included patients described in another paper included in this review (N=3)
- Study included patients who underwent ventricular infusion test and not lumbar infusion test (N=3)
- Study did not investigate the relationship between LIT and surgery outcome (N=28)
- Study did not provide sufficient data (N=2)

Included articles

(n = 10)

Additional records identified through backtracking

(n=2)

Identification

Screening

Eligibility

Inclusion

*Supplementary figure 1. PRISMA diagram of included studies*

| **Supplementary table 1. TP, FP, TN and FN of studies included in the meta-analysis** | | | | | | |
| --- | --- | --- | --- | --- | --- | --- |
|  | Cut-off for positive test | Positive outcome | TP (N) | FP (N) | TN (N) | FN (N) |
| Boon et al., 1997(1) | Rout > 18 | Any improvement on NPH Scale | 33 | 3 | 20 | 39 |
|  | Rout > 15 |  | 52 | 13 | 10 | 20 |
|  | Rout > 12 |  | 64 | 15 | 8 | 8 |
|  | Rout > 18 | Marked improvement on NPH scale | 23 | 13 | 31 | 28 |
|  | Rout > 15 |  | 36 | 29 | 15 | 15 |
|  | Rout > 12 |  | 45 | 34 | 10 | 6 |
|  | Rout > 18 | Excellent improvement on NPH scale | 11 | 25 | 41 | 18 |
|  | Rout > 15 |  | 17 | 48 | 18 | 12 |
|  | Rout > 12 |  | 24 | 55 | 11 | 5 |
| Eide et al., 2010(2) | CSF wave amplitude during infusion </> 4 mmHG | ≥2 points improvement on NPH scale | 31 | 4 | 6 | 4 |
| Junkkari et al., 2019(3) | Rout > 12 | Any improvement on NPH scale | 28 | 6 | 3 | 11 |
| Kahlon et al., 2005(4) | Rout > 18 | ≥ 2 out of 4 tests (gait, memory, spatial capacity and reaction ability) improved | 27 | 7 | 5 | 16 |
|  | Rout > 14 |  | 35 | 10 | 2 | 8 |
|  | Ppl > 22 |  | 38 | 11 | 1 | 5 |
| Raneri et al., 2017(5) | Rout > 14 | Any improvement on NPH scale | 38 | 5 | 2 | 7 |
| Sorteberg et al., 2004(6) | Rout>12 | Any improvement on NPH scale | 14 | 0 | 0 | 3 |
| Wikkelsø et al., 2013(7) | Rout > 12 | ≥5 points improvement on NPH scale | 68 | 12 | 6 | 29 |
|  | Rout > 18 |  | 30 | 2 | 16 | 67 |
| Otero‑Rodriguez et al., 2023 (8) | Rout > 12 | ≥2 points improvement on NPH scale | n/a | n/a | n/a | n/a |
|  | Rout > 13.37 |  | 69 | 12 | 24 | 17 |
|  | Rout > 15 |  | 60 | 9 | 15 | 26 |
|  | Rout > 18.39 |  | 33 | 2 | 22 | 52 |
|  | SRP > 0.03 |  | 15 | 12 | 12 | 71 |
|  | SRP > 0.043 |  | 48 | 4 | 20 | 38 |
|  | SRP > 0.047 |  | 41 | 2 | 45 | 22 |
| Van Bilsen et al., 2022(9) | Delta amplitude > 0.05 | At least one point improvement in NPH-score | 13 | 4 | 1 | 1 |
|  | Delta amplitude > 0.05 + SL |  | 19 | 6 | 7 | 6 |
|  | Delta amplitude > 0.10 |  | 10 | 4 | 1 | 4 |
|  | Delta amplitude >0.10 +SL |  | 16 | 6 | 7 | 9 |
|  | Delta amplitude > 0.15 |  | 7 | 2 | 3 | 7 |
|  | Delta amplitude > 0.15 + SL |  | 13 | 4 | 9 | 12 |
|  | Delta amplitude > 0.20 |  | 6 | 2 | 3 | 8 |
|  | Delta amplitude > 0.20 + SL |  | 13 | 4 | 9 | 12 |
|  | Delta amplitude > 0.25 |  | 2 | 1 | 4 | 12 |
|  | Delta amplitude > 0.25 + SL |  | 8 | 3 | 10 | 17 |
|  | Delta amplitude > 0.30 |  | 1 | 1 | 4 | 13 |
|  | Delta amplitude > 0.30 + SL |  | 7 | 3 | 10 | 18 |
|  | Delta amplitude > 0.35 |  | 1 | 0 | 5 | 13 |
|  | Delta amplitude > 0.35 + SL |  | 7 | 2 | 11 | 18 |
| Hasselbalch et al., 2023 (10) | Rout > 11.0 | MMSE and NPH scale (not specified further) | 79 | 18 | 5 | 25 |
|  | Rout > 15.7 |  | 53 | 13 | 10 | 51 |
|  | Rout > 18.9 |  | 24 | 8 | 15 | 80 |
|  | Pulse amplitude > 2.3 |  | 79 | 15 | 7 | 26 |
|  | Pulse amplitude > 3.6 |  | 56 | 9 | 14 | 48 |
|  | Pulse amplitude > 5.0 |  | 26 | 5 | 18 | 78 |
|  | Pulse amplitude/icp > 0.23 |  | 78 | 17 | 6 | 26 |
|  | Pulse amplitude/icp > 0.32 |  | 55 | 11 | 12 | 49 |
|  | Pulse amplitude/icp > 0.44 |  | 29 | 4 | 19 | 75 |

Rout is given in mmHg/ml/min; TP, true positives; FN, false positives; TN, true negatives; FN, false negatives; Ppl, plateau pressure in mmHg

| **Supplementary table 2. Lumbar infusion testing method of studies included in the meta-analysis** | | | | | | | |
| --- | --- | --- | --- | --- | --- | --- | --- |
|  | Patient position | Lumbar puncture needle | Baseline pressure measurement | Infusion rate | Infusion time | Aspiration of CSF | Systems used |
| Boon et al., 1997(1) | Lateral recumbent | One needle, 19-gauge | Mean value of first 5 minutes | Constant rate of 1.4 to 1.6 ml/minute | Until a stable pressure plateau was reached or the pressure exceeded 50 mm Hg | None | NR |
| Eide et al., 2010(2) | Supine | One needle, 19-gauge | Pressure directly after lumbar puncture | Constant rate of 1.5ml/minute | Not described | None | Truwave PX-600F Pressure Monitoring Set (Edwards Life sciences LLC, Irvine, CA, USA) |
| Junkkari et al., 2019(3) | Supine | Two needles, 18-gauge | Mean value of 5 minutes after the first 15-20 minutes of recording | Dependent on 7 predetermined pressure levels. If problems occurred with infusion/withdrawal in the infusion needle, an infusion rate of 1.5 mL/minute was used | Predetermined pressure levels kept at a stable level for 7 minutes each, or if no equilibrium was reached, infusion time was 20 minutes | NR | Likvor CELDA® System (CELDA, Likvor AB, Umeå, Sweden) |
| Kahlon et al., 2005(4) | Lateral recumbent | Two needles, 19-gauge | Pressure after first 10 minutes of steady state pressure | 0.80 ml/min | 45 minutes or till pressure exceeded 50mmHg | None | One of the needles was connected to a closed pressure recording device (DPT-6100, Smiths. Medical, Kirchseeon, Germany) and the other to an infusion pump (TOP syringe pump 5100; TOP Corporation, Tokyo, Japan) |
| Raneri et al., 2017(5) | Lateral recumbent | One needle, 18-gauge | Pressure during at least 10 minutes (range, 10–30 minutes) | 1.5 mL/minute in all patients with a baseline ICP <15 mm Hg | 15–35 minutes (mean, 25 minutes). The infusion was interrupted either if CSF pressure increased continuously until reaching an Ro >18 mm Hg/mL/minute or once a steady-state plateau pressure was reached | None | The needle was connected through a 3-way tap with a pressure recording device to one side and with an infusion pump to the other side (pump, Graseby 3100; recorder, Siemens Sirecust 402, Munich, Germany) |
| Sorteberg et al., 2004(6) | Lateral recumbent | One needle, 19-gauge | Before the infusion of ringer solution | 1.5 ml/min | NR | None | The needle connected to a transducer, which was linked to a computerbased system that allowed pressure sampling each 5th s. |
| Wikkelsø et al., 2013(7) | NR | One needle, 21-gauge | CSF baseline pressure was registered for 5 min before the start of LIT | NR | NR | 12 cc drainage (for CSF biomarker analyses) | NR |
| Otero‑Rodriguez et al., 2023 (8) | Lateral recumbent | One needle, 19-gauge | the basal CSF pressure (opening pressure) was recorded for 3 min before the infusion started | a constant flow rate of 1.6 ml/min. | The CSF pressure was continuously measured until the steady-state pressure was established (plateau pressure), which represented the balance between absorption and the infusion | None | The needle was connected to a 3-way tap to a closed pressure measuring device (Camino Intracranial Pressure and Temperature Monitoring™; Integra, Plainsboro, NJ, USA). This device was attached to a pressure recording system (Neuropicture™; FIMABIS, Malaga, Spain). |
| Van Bilsen et al., 2022 (9) | Supine | Two needles, 18-gauge | baseline ICP is the mean pressure during the last 5 minutes of the 15 minute baseline measurement before LIT starts | Gradual increase depending on pressure, no constant or standard flow | Gradual increase lasting 5 minutes | Pressure is reduced for 2 minutes, aiming at a total reduction of 0.8 kPa below resting ICP before the start of LIT | Likvor CELDA® System (CELDA, Likvor AB, Umeå, Sweden |
| Hasselbalch et al., 2023 (10) | Supine | Two needles, gauge not described | baseline ICP was the mean pressure during the last 5 minutes of the 15 minute baseline measurement before LIT starts | Gradual increase depending on pressure, no constant or standard flow | Gradual increase lasting 5 minutes | Pressure is reduced for 2 minutes, aiming at a total reduction of 0.8 kPa below resting ICP before the start of LIT | Likvor CELDA® System (CELDA, Likvor AB, Umeå, Sweden |

1. Boon AJ, Tans JT, Delwel EJ, Egeler-Peerdeman SM, Hanlo PW, Wurzer HA, et al. Dutch normal-pressure hydrocephalus study: prediction of outcome after shunting by resistance to outflow of cerebrospinal fluid. J Neurosurg. 1997;87(5):687-93.

2. Eide PK, Brean A. Cerebrospinal fluid pulse pressure amplitude during lumbar infusion in idiopathic normal pressure hydrocephalus can predict response to shunting. Cerebrospinal Fluid Res. 2010;7:5.

3. Junkkari A, Luikku AJ, Danner N, Jyrkkänen HK, Rauramaa T, Korhonen VE, et al. The Kuopio idiopathic normal pressure hydrocephalus protocol: initial outcome of 175 patients. Fluids Barriers CNS. 2019;16(1):21.

4. Kahlon B, Sundbarg G, Rehncrona S. Lumbar infusion test in normal pressure hydrocephalus. Acta Neurol Scand. 2005;111(6):379-84.

5. Raneri F, Zella MAS, Di Cristofori A, Zarino B, Pluderi M, Spagnoli D. Supplementary Tests in Idiopathic Normal Pressure Hydrocephalus: A Single-Center Experience with a Combined Lumbar Infusion Test and Tap Test. World Neurosurg. 2017;100:567-74.

6. Sorteberg A, Eide PK, Fremming AD. A prospective study on the clinical effect of surgical treatment of normal pressure hydrocephalus: the value of hydrodynamic evaluation. Br J Neurosurg. 2004;18(2):149-57.

7. Wikkelsø C, Hellström P, Klinge PM, Tans JT. The European iNPH Multicentre Study on the predictive values of resistance to CSF outflow and the CSF Tap Test in patients with idiopathic normal pressure hydrocephalus. J Neurol Neurosurg Psychiatry. 2013;84(5):562-8.

8. Otero-Rodriguez A, Arandia-Guzman DA, Pascual-Argente D, Ruiz-Martin L, de Oca JR, Garcia-Martin A, et al. Slope until reaching the plateau: a new predictor of valve response obtained by lumbar infusion test for idiopathic normal pressure hydrocephalus. Acta Neurochir (Wien). 2023;165(9):2533-9.

9. van Bilsen MWT, van den Abbeele L, Volovici V, Boogaarts HD, Bartels R, van Lindert EJ. The diagnostic value of the pulsatility curve to predict shunt responsiveness in patients with idiopathic normal pressure hydrocephalus. Acta neurochirurgica. 2022;164(7):1747-54.

10. Hasselbalch SG, Carlsen JF, Alaouie MM, Munch TN, Holst AV, Taudorf S, et al. Prediction of shunt response in idiopathic normal pressure hydrocephalus by combined lumbar infusion test and preoperative imaging scoring. Eur J Neurol. 2023;30(10):3047-55.
