# Supplementary material for: The diagnostic value of the lumbar infusion test to predict symptomatic improvement after shunting for normal pressure hydrocephalus. A meta-analysis
Source: Acta Neurochir (Wien). 2025 Jun 30;167(1):180. doi: 10.1007/s00701-025-06591-8 (PMC12209032; doi:10.1007/s00701-025-06591-8)
Supplement: Supplementary file 2 — (DOCX 17.5 KB) [file 701_2025_6591_MOESM2_ESM.docx]

**Supplementary Appendix 2**

*Pooled Sensitivity and pooled specificity and negative predictive values with prevalence data from included studies*

**Rout> 12**

**Summary Sensitivity**

Study | Sen [95% Conf. Iterval.] TP/(TP+FN) TN/(TN+FP)

--------------------------------------------------------------------------------------------

Boon 1997 | 0,889 0,793 - 0,951 64/72 8/23

Wikkelso 2013 | 0,701 0,600 - 0,790 68/97 6/18

Junkkari 2019 | 0,718 0,551 - 0,850 28/39 3/9

--------------------------------------------------------------------------------------------

**Pooled Sen | 0,769 0,706 - 0,825**

--------------------------------------------------------------------------------------------

Heterogeneity chi-squared = 9,76 (d.f.= 2) p = 0,008

Inconsistency (I-square) = 79,5 %

No. studies = 3.

Filter OFF

Add 1/2 to all cells of the studies with zero

**Summary Specificity**

Study | Spe [95% Conf. Iterval.] TP/(TP+FN) TN/(TN+FP)

--------------------------------------------------------------------------------------------

Boon 1997 | 0,348 0,164 - 0,573 64/72 8/23

Wikkelso 2013 | 0,333 0,133 - 0,590 68/97 6/18

Junkkari 2019 | 0,333 0,075 - 0,701 28/39 3/9

--------------------------------------------------------------------------------------------

**Pooled Spe | 0,340 0,212 - 0,488**

--------------------------------------------------------------------------------------------

Heterogeneity chi-squared = 0,01 (d.f.= 2) p = 0,994

Inconsistency (I-square) = 0,0 %

**Summary NPV**

Study | NPV [95% Conf. Iterval.] TN/(TN+FN)

--------------------------------------------------------------------------------------------

Boon 1997 | 0,500 0,247 - 0,753 8/16

Wikkelso 2013 | 0,171 0,066 - 0,336 6/35

Junkkari 2019 | 0,214 0,047 - 0,508 3/14

--------------------------------------------------------------------------------------------

**Pooled NPV (prevalence 65%) | 0,262 0,160 - 0,385**

--------------------------------------------------------------------------------------------

Heterogeneity chi-squared = 5,91 (d.f.= 2) p = 0,052

Inconsistency (I-square) = 66,1 %

**Rout> 14**

**Summary Sensitivity**

Study | Sen [95% Conf. Iterval.] TP/(TP+FN) TN/(TN+FP)

--------------------------------------------------------------------------------------------

Kahlon 2005 | 0,814 0,666 - 0,916 35/43 2/12

Raneri 2017 | 0,844 0,705 - 0,935 38/45 2/7

Rodriguez 2023 | 0,802 0,702 - 0,880 69/86 12/24

--------------------------------------------------------------------------------------------

**Pooled Sen | 0,816 0,750 - 0,871**

--------------------------------------------------------------------------------------------

Heterogeneity chi-squared = 0,36 (d.f.= 2) p = 0,836

Inconsistency (I-square) = 0,0 %

No. studies = 3.

Filter OFF

Add 1/2 to all cells of the studies with zero

**Summary Specificity**

Study | Spe [95% Conf. Iterval.] TP/(TP+FN) TN/(TN+FP)

--------------------------------------------------------------------------------------------

Kahlon 2005 | 0,167 0,021 - 0,484 35/43 2/12

Raneri 2017 | 0,286 0,037 - 0,710 38/45 2/7

Rodriguez 2023 | 0,500 0,291 - 0,709 69/86 12/24

--------------------------------------------------------------------------------------------

**Pooled Spe | 0,372 0,230 - 0,533**

--------------------------------------------------------------------------------------------

Heterogeneity chi-squared = 4,31 (d.f.= 2) p = 0,116

Inconsistency (I-square) = 53,5 %

--------------------------------------------------------------------------------------------

**Summary NPV**

Study | NPV [95% Conf. Iterval.] TN/(TN+FN)

--------------------------------------------------------------------------------------------

Kahlon 2005 | 0,200 0,025 - 0,556 2/10

Raneri 2017 | 0,222 0,028 - 0,600 2/9

Rodriguez 2023 | 0,414 0,235 - 0,611 12/29

--------------------------------------------------------------------------------------------

**Pooled NPV (Prevalence 65%) | 0,333 0,204 - 0,484**

--------------------------------------------------------------------------------------------

Heterogeneity chi-squared = 2,23 (d.f.= 2) p = 0,328

Inconsistency (I-square) = 10,2 %

**Rout> 18**

**Summary Sensitivity**

Study | Sen [95% Conf. Iterval.] TP/(TP+FN) TN/(TN+FP)

--------------------------------------------------------------------------------------------

Boon 1997 | 0,458 0,340 - 0,580 33/72 20/23

Kahlon 2005 | 0,628 0,467 - 0,770 27/43 5/12

Wikkelso 2013 | 0,309 0,219 - 0,411 30/97 16/18

Rodriguez 2023 | 0,384 0,281 - 0,495 33/86 22/24

Hasselbach 2023 | 0,231 0,154 - 0,324 24/104 15/23

--------------------------------------------------------------------------------------------

**Pooled Sen | 0,366 0,318 - 0,415**

--------------------------------------------------------------------------------------------

Heterogeneity chi-squared = 24,96 (d.f.= 4) p = 0,000

Inconsistency (I-square) = 84,0 %

No. studies = 5.

Filter OFF

Add 1/2 to all cells of the studies with zero

**Summary Specificity**

Study | Spe [95% Conf. Iterval.] TP/(TP+FN) TN/(TN+FP)

--------------------------------------------------------------------------------------------

Boon 1997 | 0,870 0,664 - 0,972 33/72 20/23

Kahlon 2005 | 0,417 0,152 - 0,723 27/43 5/12

Wikkelso 2013 | 0,889 0,653 - 0,986 30/97 16/18

Rodriguez 2023 | 0,917 0,730 - 0,990 33/86 22/24

Hasselbach 2023 | 0,652 0,427 - 0,836 24/104 15/23

--------------------------------------------------------------------------------------------

**Pooled Spe | 0,780 0,686 - 0,857**

--------------------------------------------------------------------------------------------

Heterogeneity chi-squared = 15,22 (d.f.= 4) p = 0,004

Inconsistency (I-square) = 73,7 %

**Summary NPV**

Study | NPV [95% Conf. Iterval.] TN/(TN+FN)

--------------------------------------------------------------------------------------------

Boon 1997 | 0,339 0,221 - 0,474 20/59

Kahlon 2005 | 0,238 0,082 - 0,472 5/21

Wikkelso 2013 | 0,193 0,114 - 0,294 16/83

Rodriguez 2023 | 0,293 0,194 - 0,410 22/75

Hasselbach 2023 | 0,158 0,091 - 0,247 15/95

--------------------------------------------------------------------------------------------

**Pooled NPV (Prevalence 65%) | 0,234 0,190 - 0,283**

--------------------------------------------------------------------------------------------

Heterogeneity chi-squared = 8,90 (d.f.= 4) p = 0,064

Inconsistency (I-square) = 55,1 %
